# Office Blood Pressure and Obesity in Children with X-Linked Hypophosphatemia

**DOI:** 10.1007/s00223-025-01363-z

**Published:** 2025-03-28

**Authors:** Ineke Böckmann, Maren Leifheit-Nestler, Mirko Rehberg, Giuseppina Spartà, Katrina Evers, Karl Peter Schlingmann, Markus J. Kemper, Annette Richter-Unruh, Olaf Hiort, Karina Grohmann-Held, Ute Derichs, Clemens Freiberg, Marcus Weitz, Desiree Dunstheimer, Elmar Schmid, Ulrike John-Kroegel, Oliver Metzing, Sabine Heger, Norbert Jorch, Hagen Staude, Ludwig Patzer, Elke Wühl, Miroslav Zivicnjak, Dirk Schnabel, Dieter Haffner

**Affiliations:** 1 Department of Pediatric Kidney, Liver, Metabolic and Neurological Diseases, Hannover Medical School, Carl-Neuberg-Str. 1, 30625 Hannover, Germany; 2 Department of Pediatrics, University of Cologne, Cologne, Germany; 3 Pediatric Nephrology, University Children’s Hospital Zurich, Zurich, Switzerland; 4 Department of General Pediatrics, Pediatric Nephrology, University Children’s Hospital, Münster, Germany; 5 Asklepios Children’s Hospital Hamburg-Heidberg, Hamburg, Germany; 6 University Children’s Hospital Bochum, Bochum, Germany; 7 Division of Pediatric Endocrinology and Diabetes, Department of Pediatric and Adolescent Medicine, University of Lübeck, Lübeck, Germany; 8 University Children’s Hospital Greifswald, Greifswald, Germany; 9 University Children’s Hospital, Mainz, Germany; 10 Department of Pediatrics, University Medicine Göttingen, Göttingen, Germany; 11 Department of General Pediatrics and Hematology/Oncology, University Children’s Hospital, University Hospital Tübingen, Tübingen, Germany; 12 University Children’s Hospital Augsburg, Augsburg, Germany; 13 Pediatric Practice Dres. Schmid, Bettendorf, Hammon & Zimmermann, Hirschaid, Germany; 14 Department of Pediatric Nephrology, University Children’s Hospital, Jena, Germany; 15 Department of Pediatric Endocrinology, University Children’s Hospital, Jena, Germany; 16 Kinderkrankenhaus Auf Der Bult, Hannover, Germany; 17 University Children’s Hospital, Evangelisches Klinikum Bethel, Bielefeld, Germany; 18 University Children’s Hospital Rostock, Rostock, Germany; 19 St. Elisabeth and St. Barbara Children’s Hospital, Halle/Saale, Germany; 20 Division of Pediatric Nephrology, Medical Faculty Heidelberg, Center for Pediatrics and Adolescent Medicine, Heidelberg University Hospital, Heidelberg, Germany; 21 Center for Chronically Sick Children, Pediatric Endocrinology, University Medicine, Charité Berlin, Berlin, Germany

**Keywords:** X-linked hypophosphatemia, Hypertension, Blood pressure, Body mass index, Burosumab

## Abstract

X-linked hypophosphatemia (XLH) is the most common inherited form of hypophosphatemic rickets. Children with XLH have an increased risk of obesity, which may promote high blood pressure, but data on blood pressure in XLH are inconclusive. We aimed to assess blood pressure and its determinants in pediatric XLH patients. We conduct a prospective, multicenter observational study of children with XLH in Germany and Switzerland. Office blood pressure and body mass index (BMI) were annually measured in 128 pediatric XLH patients with a median follow-up of 2 years (range 1–6). Potential predictors of blood pressure were investigated by Spearman correlation. Seventeen percent of patients were treated with phosphate supplements and active vitamin D for a median of 8 years, 83% of patients received burosumab for 2.3 years with 3.1 years of prior treatment with phosphate supplements and active vitamin D. Median systolic (0.75 z-score) and diastolic (0.32 z-score) blood pressure and BMI (0.72 z-score) were increased compared to healthy children (each* p* < 0.01). The prevalence of obesity (9.8% vs. 3%), arterial hypertension (26.2% vs. 5%), and high-normal blood pressure (22.9% vs. 5%) was higher in the XLH cohort compared to the general pediatric population (each *p* < 0.001). Spearman rank correlation analysis revealed significant associations between both systolic (r = 0.24; *p* < 0.01) and diastolic (r = 0.20; *p* < 0.05) blood pressure with BMI, while the mode of treatment, i.e. burosumab versus phosphate supplements and active vitamin D, was no significant correlate. Children with XLH present with elevated office blood pressure values, associated with elevated BMI.

**Supplementary Information:**

The online version contains supplementary material available at 10.1007/s00223-025-01363-z.

## Introduction

X-linked hypophosphatemia (XLH) is the most common genetic cause of hypophosphatemic rickets [1] with a prevalence of 1.7–4.8/100,000 children [2, 3]. Pathogenic variants in the *PHEX* (phosphate-regulating neutral endopeptidase homolog X-linked) gene lead to an increased secretion of the phosphaturic hormone fibroblast growth factor 23 (FGF23) from bone, consecutive renal phosphate wasting and impaired synthesis of 1,25-dihydroxyvitamin D_3_ (1,25(OH)_2_D_3_), resulting in hypophosphatemia [4]. Children with XLH usually present with rickets, osteomalacia, bone pain, disproportionate short stature within the first two years of life, and later on with tooth abscesses [5, 6].

Treatment of XLH with frequent oral phosphate supplements and active vitamin D has limited efficacy in healing of rickets, and is associated with adverse effects including nephrocalcinosis and hyperparathyroidism [7, 8]. Burosumab, a monoclonal anti-FGF23 antibody, was shown to be superior to treatment with phosphate and active vitamin D in healing of rickets and growth, and safe in terms of adverse effects [9].

Experimental studies and clinical studies in patients with chronic kidney disease (CKD) and the general population have shown that FGF23 induces left ventricular hypertrophy by activating the PLCγ/calcineurin/NFAT pathway via FGF receptor 4 independent of the co-receptor α-Klotho [10, 11] and causes arterial stiffness via enhanced FGF receptor-dependent reactive oxygen species (ROS) production [12, 13]. As FGF23 levels are elevated in XLH patients and further stimulated by treatment with phosphate supplements and active vitamin D, interest in the cardiovascular risk profile of XLH patients arose [14]. XLH patients were found to have an unfavorable body composition with increased body fat percentage and decreased muscle mass [15] with up to 60% of XLH patients being classified as overweight which may promote hypertension in these patients [16–18]. Some clinical studies found increased office blood pressure values in adults and children with XLH [17, 19, 20], others reported normal blood pressure values [15, 21, 22]. These conflicting results may at least be partly due to the low number of patients investigated.

We hypothesized that, (i) office blood pressure is increased in children and adolescents with XLH, and (ii) associated with the degree of obesity. Therefore, we analyzed blood pressure and BMI in 128 German and Swiss children with XLH on treatment with phosphate supplements and active vitamin D or burosumab being followed up in a prospective, binational, multicenter registry and observational study.

## Methods

### Patients and Study Design

The present work is an analysis of the prospective, binational, multicenter registry and observational study “Growth and comorbidity in children with XLH” by the German Society for Pediatric Nephrology (GPN) and the German Society for Pediatric and Adolescent Endocrinology and Diabetology (DGPAED), which was initiated in 2018 [23]. The study/registry was approved by the ethics committee of the Institutional Ethics Review Board at Hannover Medical School (No. 7259) and from each participating center and was performed according to the Declaration of Helsinki. Patients and/or parents/guardians provided written informed consent and assent to participate in the study.

The study included patients aged 0–18 years diagnosed with XLH based on family history and/or genetic confirmation, presence of clinical and/or radiological signs of rickets, impaired height velocity and serum phosphate levels below the age-related reference range, associated with selective renal phosphate wasting in the absence of vitamin D or calcium deficiency (9). The diagnosis of XLH was genetically proven in all patients with a negative family history. From March 2018 until August 2024, 147 patients were enrolled in 40 participating centers in Germany and Switzerland.

**Fig. 1:**
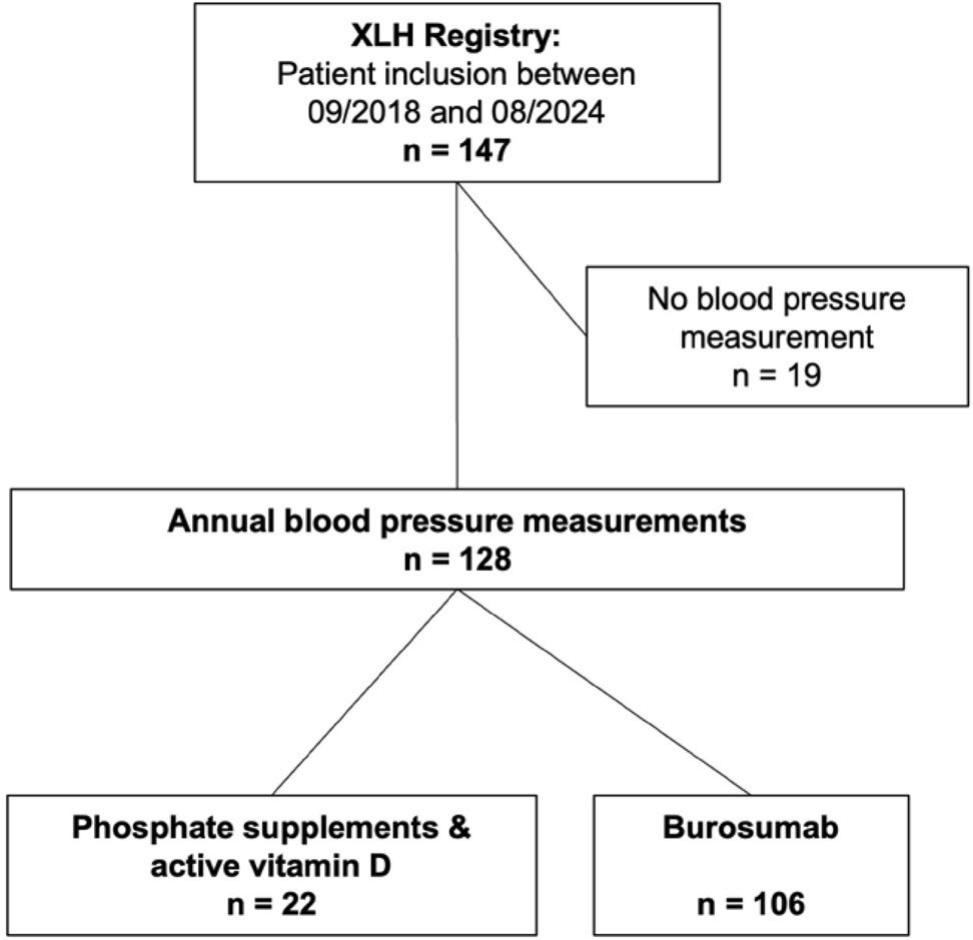
The patient cohort: Annual blood pressure measurements were available in 128 out of the 147 patients included in the XLH registry, with a median follow-up of 2 years (range 6). Of these, 22 were treated with phosphate supplements and active vitamin D, and 106 were on burosumab treatment

This analysis is restricted to 128 XLH patients (72 girls) with available office blood pressure measurements (Fig. 1). We collected the following data: age, sex, family history, result of *PHEX* gene analysis, age at diagnosis and start of treatment, concomitant medication, systolic and diastolic blood pressure, weight, height, presence of nephrocalcinosis in renal ultrasound, serum phosphate, alkaline phosphatase (ALP), intact parathyroid hormone (PTH) and creatinine. Physicians were required to comply with EMA (European Medicines Agency) recommendations on burosumab treatment in pediatric XLH patients from 2018 [24] and its revised version from 2022 [25]. In the latter, a higher starting dose of burosumab, i.e. 0.8 mg/kg body weight instead of 0.4 mg/kg per two weeks, was recommended.

### Methods

Office blood pressure was annually measured oscillometrically after a phase of physical inactivity, as recommended [26], with a median follow-up of 2 years (range 1–6). Arterial hypertension and high-normal blood pressure were defined as systolic and/or diastolic blood pressure of ≥ the 95th percentile and ≥ the 90th to < the 95th percentile for sex, age and height, respectively [26]. For calculation of body mass index (BMI), we used the following equation: BMI = weight (kg) / height (m)^2^. Overweight, obesity and extreme obesity were defined as BMI values > the 90th to ≤ the 97th percentile, > the 97th percentile, and > the 99.5th percentile, respectively [27]. We used national reference values of healthy children for anthropometric and blood pressure parameters to calculate patient z-scores taking into account age, sex and height [28, 29]. The reference values determined by Kromeyer-Hauschild et al. in 2001 (data acquisition 1985–1999) [29] were used for children aged ≥ 2 years to avoid the confounding effect of the German obesity epidemic that is already present in the data of Neuhauser et al. (data acquisition 2003–2006) [28].

We compared the prevalence of high-normal blood pressure, hypertension, overweight and obesity in our patient cohort to the corresponding prevalence in the German general pediatric population [28]. For serum phosphate levels, we used German reference values from the HAnnover Reference values for Pediatrics (HARP) study [30] to calculate z-scores. Z-scores were also calculated for ALP (considering the local methodology) [31], and PTH [32] using appropriate reference values. The estimated glomerular filtration rate (eGFR) was calculated using the revised Schwartz formula [33].

### Statistical Analysis

Data is presented as median (interquartile range, IQR) or n (%), as appropriate. The median value of office blood pressure in each patient was used to calculate individual blood pressure z-scores. Only patients with at least three annual blood pressure measurements were included to classify patients as being hypertensive or showing high normal blood pressure (*n* = 61). Ten and 51 of these patients were treated with phosphate supplements and active vitamin D, and burosumab, respectively. Differences between our study population and healthy children were analyzed using z-scores and Wilcoxon-tests. Differences between the groups were analyzed using Mann–Whitney-U-test or Chi-squared-Test, respectively. Spearman-Rho’s correlation analyses were used to investigate associations between blood pressure values and potential predictors, including age, age at diagnosis, age at start of treatment, sex, current treatment modality, duration of treatment with phosphate supplements and active vitamin D or burosumab, dosage of active/native vitamin D, phosphate supplements, and burosumab, anthropometric parameters, presence of nephrocalcinosis, serum 25OHD, z-scores of serum phosphate, ALP and PTH, and eGFR. Statistical significance was defined by *p* values < 0.05. SPSS Statistics version 29.0.1 for Windows RRID:SCR_002865 (IBM Corporation, New York, NY) and GraphPad Prism version 10.2 for Windows RRID:SCR_002798 (GraphPad Software, Boston, Massachusetts USA) were used.

## Results

### Patient Characteristics

The median age at diagnosis and first clinical observation was 1.1 (IQR 0.2–2.5) and 10.3 (IQR 5.4–14.1) years respectively (Table 1). Twenty-two patients (17%) were on treatment with phosphate supplements and active vitamin D for a median period of 8.0 years (IQR 2.4–11.0), 106 patients (83%) received burosumab for a median period of 2.3 years (IQR 1.1–3.2) with 87/106 (82%) patients receiving prior treatment with phosphate supplements and active vitamin D over a period of 3.1 years (IQR 0.7–8.5), while 19/106 (18%) patients were primarily started on burosumab. Therapy with phosphate supplements and active vitamin D consisted of 21 mg/kg/day (IQR 10.8–29.1) elemental phosphorus and 11.7 ng/kg/day (IQR 4.0–28.0) calcitriol. The median dosage of burosumab amounted to 0.6 mg/kg (IQR 0.44–1.11) every 14 days. None of the patients received antihypertensive medication. Native vitamin D (cholecalciferol) was administered more frequently to patients on treatment with phosphate supplements and active vitamin D compared to burosumab (23.3% versus 5.1%, *p* < 0.01). The median 25OHD level was 25.3 ng/ml and 22.7% of patients were vitamin D insufficient, there was no difference between the two treatment groups.

**Table 1:** Clinical characteristics of 128 pediatric XLH patients on treatment with phosphate supplements and active vitamin D or burosumab

Characteristics	All patients	Phosphate and active vitamin D	Burosumab	*p*
n	128 (100)	22 (17)	106 (83)	n.a
Female, n	72 (56)	12 (17)	60 (83)	0.753
Age at diagnosis, years	1.1 (0.2–2.5)	0.8 (0.3–2.5)	1.2 (0.1–2.6)	0.842
Age at observation, years	10.3 (5.4–14.1)	11.2 (6.3–14.2)	10.2 (5.3–14.1)	0.410
Height, cm	129.6 (105.1–149.0)	136.5 (105.1–149.6)	128.6 (105.1–149.4)	0.477
Height, z-score	− 1.92 (− 2.65 to − 1.22)^a^	− 1.53 (− 2.63 to − 0.90)^a^	− 1.96 (− 2.68 to − 1.28)^a^	0.157
Body weight, kg	30.1 (18.5; 51)	33.2 (22.1–50.9)	29.7 (18.2–51.3)	0.654
Body weight, z-score	− 0.70 (− 1.29–0.12)^a^	− 0.54 (− 1.30–0.16)	− 0.72 (− 1.31–0.15)^a^	0.784
P + vit.D treatment, years	n.a	8.0 (2.4–11.0)	n.a	n.a
Burosumab, years	n.a	n.a	2.3 (1.1–3.2)	n.a
Prior P + vit.D treatment, years	n.a	n.a	3.1 (0.7–8.5)	n.a
Phosphorus, mg/kg/day	n.a	21.0 (10.8–29.1)	n.a	n.a
Calcitriol, ng/kg/day	n.a	11.7 (4.0–28.0)	n.a	n.a
Burosumab, mg/kg/14 days	n.a	n.a	0.6 (0.4–1.1)	n.a
Serum phosphate, mmol/l	1.00 (0.87 − 1.13)	0.90 (0.72–1.00)	1.03 (0.88–1.16)	0.004
Serum phosphate, z-score	− 2.78 (− 3.57 to − 1.99)^a^	− 3.34 (− 4.42 to − 2.51)^a^	− 2.68 (− 3.51 to − 1.86)^a^	0.036
Serum ALP, U/l	336 (252–432)	402 (282–508)	329 (249–412)	0.060
Serum ALP, z-score	1.73 (0.77–2.78)^a^	2.36 (1.67–3.79)^a^	1.47 (0.72–2.51)^a^	0.007
Serum PTH, ng/l	46.4 (34.6–69.8)	73.7 (36.4–96.5)	44.3 (33.3–61.8)	0.037
Serum PTH, z-score	1.24 (0.44–2.35)^a^	2.50 (0.58–3.24)^a^	1.11 (0.34–2.02)^a^	0.037
25OHD, ng/ml	25.3 (20.0–32.8)	23.8 (16.8–32.6)	23.5 (20.5–29.3)	0.368
25OHD < 30 ng/ml, n	81 (63.3)	12 (54.5)	69 (65.1)	0.537
25OHD < 20 ng/ml, n	29 (22.7)	7 (31.8)	22 (20.8)	0.163
eGFR, ml/min/1.73m^2^	127 (106–144)	124 (96–138)	127 (106–144)	0.493

Data is presented as median (interquartile range) or n (%), *p* values were calculated using unpaired Mann–Whitney test or Chi-squared-Test, respectively

n.a., not applicable; P + vit.D, phosphate supplements and active vitamin D; eGFR, estimated glomerular filtration rate; ALP, alkaline phosphatase; PTH, parathyroid hormone

^a^*p* < 0.001 versus healthy controls

Standardized height and weight were significantly reduced in both groups. Median serum phosphate z-scores were significantly higher in patients on burosumab compared to treatment with phosphate supplements and active vitamin D therapy (− 2.68 (IQR − 3.51 to − 1.86) versus − 3.34 (IQR − 4.42 to − 2.51), *p* < 0.05) (55, 56). Serum ALP z-scores were significantly higher in children treated with phosphate supplements and active vitamin D compared to those on burosumab (2.36 (IQR 1.67–3.79) versus 1.47 (IQR 0.72–2.51), *p* < 0.01). PTH levels were elevated compared to healthy children, and more pronounced in patients treated with phosphate supplements and active vitamin D compared to those on burosumab (2.50 z-score (IQR 0.58–3.24) versus 1.11 z-score (IQR 0.34–2.02), *p* < 0.05). eGFR was normal, irrespective of treatment modality. The prevalence of nephrocalcinosis did not differ between groups (phosphate supplements and active vitamin D treatment 36%, burosumab 30%, *p* = 0.7520).

### Blood Pressure

**Fig. 2:**
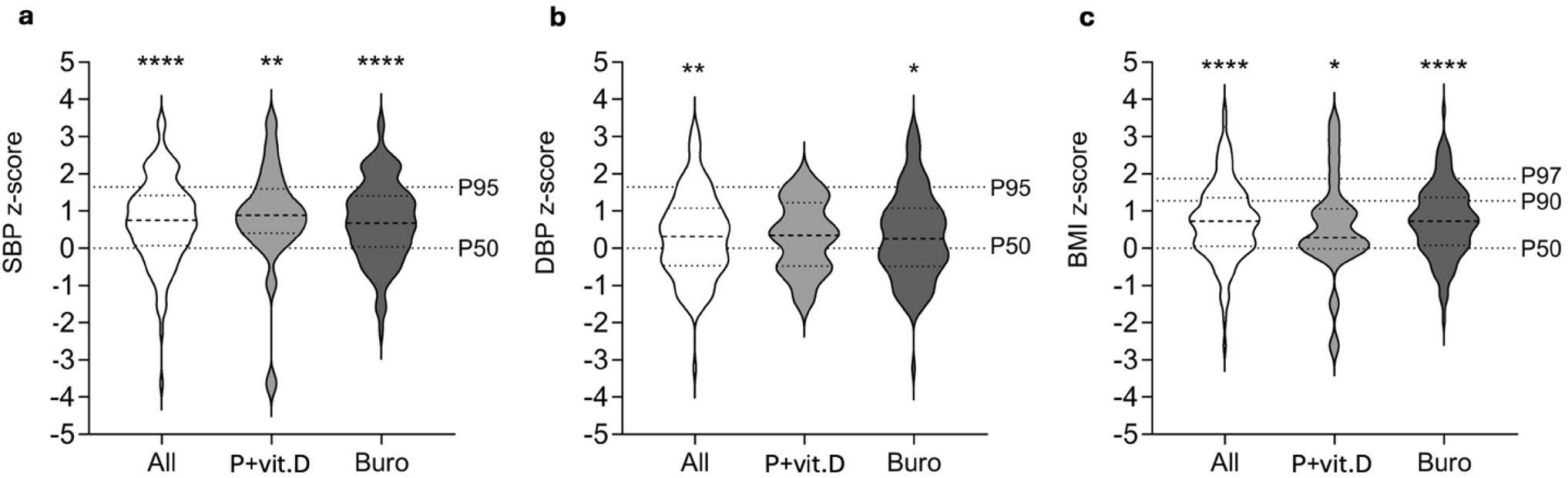
Systolic blood pressure (**a**), diastolic blood pressure (**b**) and body mass index (**c**) z-scores in pediatric XLH patients on treatment with phosphate supplements and vitamin D or burosumab. *p < 0.05 versus healthy children; **p < 0.01 versus healthy children; ****p < 0.001 versus healthy children. P + vit.D, phosphate supplements and active vitamin D; *Buro* burosumab, *SBP* systolic blood pressure, *DBP* diastolic blood pressure, *BMI* body mass index

Median systolic and diastolic blood pressure z-scores in children with XLH were significantly elevated compared to healthy children by 0.75 SD (IQR 0.07–1.42; *p* < 0.001) and 0.32 SD (IQR − 0.47 to 1.08;* p* < 0.01), respectively (Table 2**, **Fig. 2). Systolic and diastolic blood pressure appeared to be higher in the patients on phosphate supplements and active vitamin D compared to those on burosumab (0.89 z-score (IQR 0.41–1.60) vs 0.68 z-score (IQR 0.04–1.41), *p* = 0.396, and 0.35 z-score (IQR − 0.48 to 1.24) vs 0.25 z-score (IQR − 0.49 to 1.08), *p* = 0.985) but the difference did not reach the level of statistical significance.

**Table 2:** Blood pressure and body mass index in 128 pediatric XLH patients on treatment with phosphate supplements and active vitamin D or burosumab

Characteristics	All (n = 128)	Phosphate and active vitamin D (*n* = 22)	Burosumab (*n* = 106)	*p*
Median systolic BP, mmHg	110.5 (103.0–120.5)	111.0 (107.5–121)	110 (102.8–120.5)	0.501
Median systolic BP, z-score	0.75 (0.07–1.42)^b^	0.89 (0.41–1.60)^a^	0.68 (0.04–1.41)^b^	0.396
Median diastolic BP, mmHg	66.0 (60.0–71.5)	67.3 (60.8–71.3)	66.0 (60.0–71.5)	0.646
Median diastolic BP, z-score	0.32 (− 0.47–1.08)^a^	0.35 (− 0.48–1.24)	0.25 (− 0.49–1.08)^a^	0.985
Median BMI, kg/m^2^	25.9 (18.6–35.7)	27.6 (20.1–35.8)	24.6 (18.2–36.1)	0.628
Median BMI, z-score	0.72 (0.05–1.36)^b^	0.37 (− 0.09–1.07)^a^	0.75 (0.07–1.37)^b^	0.468

Data is presented as median (interquartile range), *p* values were calculated using unpaired Mann–Whitney test

*BP* blood pressure, *BMI* body mass index

^a^*p* < 0.05 versus healthy controls

^b^*p* < 0.001 versus healthy controls

**Fig. 3:**
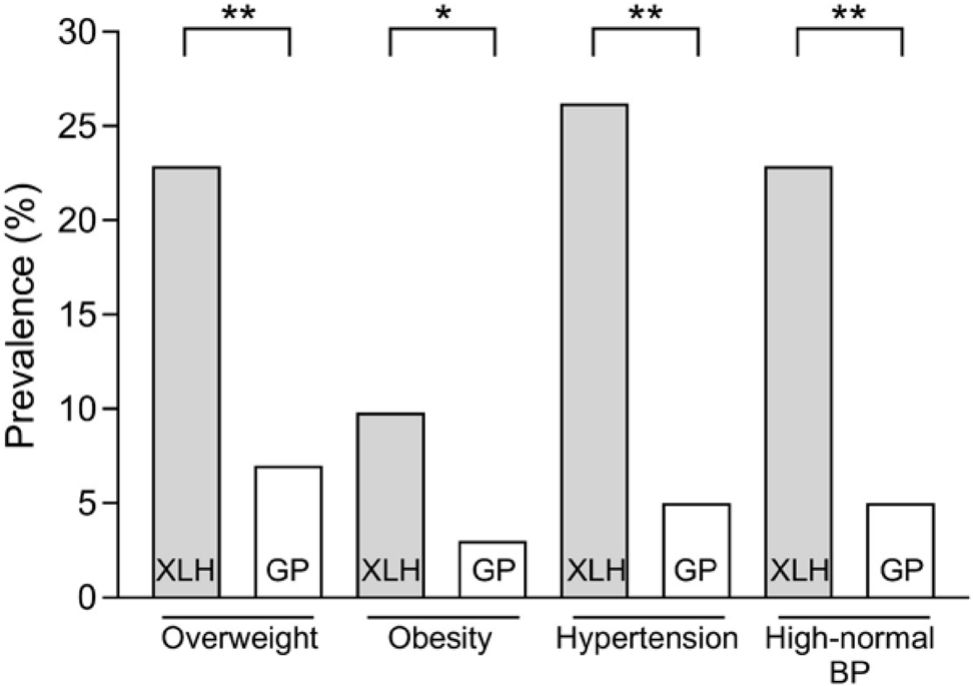
Prevalence of overweight, obesity, hypertension and high-normal blood pressure in the XLH patient cohort versus the German general pediatric population [28]. *P* values were calculated using Chi-squared test. *p < 0.01; **p < 0.001. *XLH* X-linked hypophosphatemia, *GP* German general pediatric population, *BP* blood pressure

Arterial hypertension and high-normal blood pressure were found to be significantly more prevalent in our XLH population compared to the German general pediatric population (hypertension: 26.2% versus 5%; high-normal blood pressure: 22.9% versus 5%; each *p* < 0.001) (Table 3**, **Fig. 3). High-normal blood pressure and hypertension seemed to be more prevalent in the group treated with phosphate supplements and active vitamin D compared to those on burosumab but this did not reach the level of statistical significance (*p* = 0.217 and *p* = 0.110).

**Table 3:** Prevalence of high-normal blood pressure, hypertension, overweight, obesity and extreme obesity in 61 pediatric XLH patients on treatment with phosphate supplements and active vitamin D or burosumab with at least 3 annual blood pressure measurements

Characteristics	All (*n* = 61)	Phosphate and active vitamin D (*n* = 10)	Burosumab (*n* = 51)	*p*
High-normal BP, n	14 (22.9)	4 (40)	10 (19.6)	0.217
Hypertension, n	16 (26.2)	5 (50)	11 (21.6)	0.110
Overweight, n	14 (22.9)	1 (10)	13 (25.5)	0.429
Obesity, n	6 (9.8)	2 (20)	4 (7.8)	0.253
Of which extreme obesity, n	3 (4.9)	1 (10)	2 (3.9)	0.421

Data is presented as n (%), *p* values were calculated using Chi-squared test

Children < 16 years: high-normal BP = systolic and/or diastolic BP ≥ 90th to < 95th percentile, hypertension = systolic and/or diastolic BP ≥ 95th percentile

Children ≥ 16 years: high-normal BP = 130/85 to 139/90 mmHg; hypertension =  ≥ 140/90 mmHg

Overweight = BMI > 90th to ≤ 97th percentile, obesity = BMI > 97th percentile, extreme obesity = BMI > 99.5th percentile

*BP* blood pressure

### Body Mass Index

Median BMI (0.72 z-score (IQR 0.05–1.36)) was elevated compared to healthy children (*p* < 0.001), which tended to be more pronounced in patients on burosumab treatment (0.75 z-score (IQR 0.07–1.37), *p* < 0.001 versus healthy children) compared to those on treatment with phosphate supplements and active vitamin D (0.37 z-score (IQR − 0.09 to 1.07), *p* < 0.05 versus healthy children; *p* = 0.468 versus burosumab group) (Table 2**, **Fig. 2). Likewise, the prevalence of overweight tended to be higher in patients on burosumab compared to those on phosphate supplements and active vitamin D (25.5% versus 10%, *p* = 0.429), while the prevalence of obesity tended to be less frequent in the burosumab compared to the phosphate and active vitamin D group (7.8% versus 20%, *p* = 0.253). The prevalence of overweight and obesity in our patient cohort was significantly higher compared to the German general pediatric population (22.9% versus 7%, p < 0.001 and 9.8% versus 3%, *p* < 0.01) (Fig. 3).

### Predictors of Office Blood Pressure

**Fig. 4:**
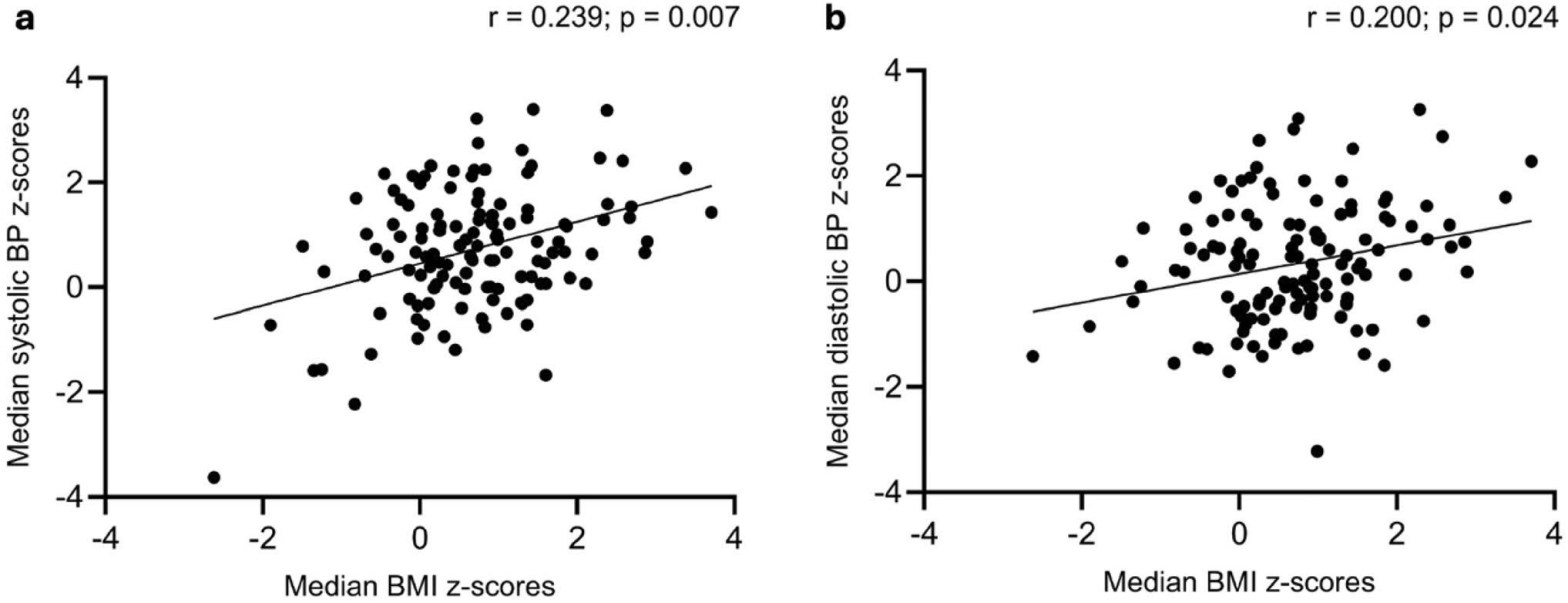
Systolic (**a**) and diastolic (**b**) blood pressure as a function of BMI in 128 pediatric XLH patients on treatment with phosphate supplements and active vitamin D or burosumab. A: *r* = 0.239, *p* = 0.007; B: *r* = 0.200, *p* = 0.024. BP, blood pressure; BMI, body mass index

Systolic (*r* = 0.239, *p* = 0.007) and diastolic (*r* = 0.200, *p* = 0.024) blood pressure z-scores were significantly associated with BMI z-score (Fig. 4). Likewise, body weight and height z-scores were associated with systolic and diastolic blood pressure z-scores (weight and systolic blood pressure z-scores: *r* = 0.259, *p* = 0.003; height and systolic blood pressure z-scores: *r* = 0.186, *p* = 0.035; weight and diastolic blood pressure z-scores: *r* = 0.261, *p* = 0.003; height and diastolic blood pressure: *r* = 0.196, *p* = 0.027). All other potential predictors of blood pressure included in our analysis were no significant correlates, including presence of nephrocalcinosis and treatment modality. Likewise, the comparison of other parameters than BMI z-score in patients with and without hypertension revealed no significant differences between groups (Supplementary Table 1).

## Discussion

This is the largest study on blood pressure values in patients with XLH. Systolic and diastolic office blood pressure as well as BMI values were significantly increased in pediatric XLH patients compared to healthy children by approximately 0.8 z-score, 0.3 z-score, and 0.7 z-score, respectively. Similarly, the prevalence of hypertension (26.2% versus 5%), high-normal blood pressure (22.9% versus 5%), and obesity (9.8% vs. 3%) in our XLH cohort was significantly higher compared to the German general pediatric population. Blood pressure values were significantly associated with the degree of obesity while the mode of treatment, i.e. phosphate supplements and active vitamin D versus burosumab, was no significant correlate. This study highlights the need for cardiovascular risk monitoring in children with XLH.

Obesity and high blood pressure are the most important risk factors for the development of cardiovascular disease in children [34, 35]. Blood pressure is highly associated with obesity and secular trends in obesity were documented in Western pediatric populations probably caused by changes in dietary intake and physical activity during the last 3 decades [28]. A high prevalence of obesity and/or overweight was previously reported in patients with XLH, with approximately one in three pediatric and two in three adult patients affected [16–18]. The respective mean BMI z-scores in pediatric XLH patients ranged between 0.6 and 2.0 and were associated with age, treatment duration, and positive family history for XLH [6, 36]. In the present study, both the mean BMI (0.72 z score) and the prevalence of obesity or overweight (26.2%) were at the lower end compared to the values reported in the above-mentioned studies in children with XLH. This discrepancy is at least partly due to the rather mild phenotypes observed in patients who continued to be treated with phosphate supplements and active vitamin D in the present study despite the general availability of burosumab for children with XLH in Germany and Switzerland. Indeed, patients remaining on phosphate supplements and active vitamin D showed only a mild degree of short stature (-1.53 z-score) and only moderately increased ALP levels (2.36 z-score) despite rather low dosages of phosphate supplements (median of 21 mg/kg/d) compared to previous reports (around 40 mg/kg/day) in pediatric XLH patients on phosphate supplements and active vitamin D [1, 23]. In addition, since burosumab is known to improve physical performance, patients treated with burosumab in the present study may be able to perform more physical activities in their daily lives compared to those in previous reports on blood pressure, mostly including children on phosphate supplements and active vitamin D treatment [37]. Nevertheless, the prevalence of obesity was significantly increased compared to the German general pediatric population (9.8% versus 3%) supporting the concept that pediatric XLH patients are at increased risk of obesity.

Systolic and diastolic blood pressure values in our XLH cohort were significantly elevated by 0.8 SD and 0.3 SD compared to healthy children. The prevalence of arterial hypertension (26.2% versus 5.0%) and high-normal blood pressure (22.9% versus 5.0%) was significantly higher in our XLH population compared to the German general pediatric population. It was noticeable that both systolic and diastolic blood pressure values tended to be around 0.2 SD and 0.1 SD, respectively, higher in children on treatment with phosphate supplements and active vitamin D compared to those on burosumab, although the latter showed higher BMI z-scores (0.75 versus 0.37). In the regression analyses we noted that blood pressure values were associated with BMI z-scores, which is in line with previous studies evaluating blood pressure values in children [28, 34, 35]. Therefore, the higher BMI values in children on burosumab may have blunted differences in blood pressure z-scores between children on phosphate supplements and vitamin D compared to those on burosumab in the present study. The impact of treatment modality should be investigated in future studies including larger numbers of patients under treatment with phosphate supplements and active vitamin D in comparison to burosumab treatment.

In principle, elevated blood pressure in children with XLH may be related to the nature of the disease itself and/or to the treatment. In untreated *Hyp* mice, the animal model of XLH, an increased systolic and diastolic blood pressure and mean arterial pressure has been shown [38, 39]. However, subsequent studies in *Hyp* mice could not demonstrate any pathological cardiac remodeling [40]. It was assumed that FGF23 levels in *Hyp* mice are below the threshold required for left ventricular hypertrophy development or that hypophosphatemia may prevent the deleterious effects of FGF23 on the heart [40].

Studies in adult XLH patients, including 22–24 patients, have shown a prevalence of hypertension of 9%–45%, but no left ventricular hypertrophy [19, 20]. Corresponding pediatric studies including 11–41 patients found conflicting results. While some studies report elevated blood pressure compared to controls or hypertension by definition [17, 20], Vered et al. and Brener et al. each found no hypertension in their patient cohorts [15, 21]. Nehgme et al. noted left ventricular hypertrophy in 10 of their 13 patients [41], while all other studies mentioned above showed no or only very rare left ventricular hypertrophy.

Experimental studies have shown significant effects of FGF23 on the vascular system. Firstly, administration of FGF23 in healthy mice and elevated FGF23 levels in *Hyp* mice results in activation of the sodium chloride cotransporter in the distal tubule, leading to reabsorption of sodium, chloride and water and consequently to elevated blood pressure [39]. Secondly, there is increasing evidence that elevated FGF23 causes vascular stiffness. In in vitro experiments, high levels of FGF23 in the supernatant resulted in increased vascular wall thickness and stiffening as well as an increased production of ROS in vascular smooth muscle cell culture and led to contraction of mouse aorta rings [12, 42]. Similarly, increased peripheral vascular resistance was demonstrated in *Hyp* mice [38]. A recent meta-analysis of 62 clinical studies revealed an association of FGF23 and its cofactor Klotho with arterial calcification, vascular wall thickness and stiffness [13]. This association was stronger in CKD patients, but still significant in non-CKD individuals. Thirdly, wild type mice on a high phosphate diet show an increase in serum FGF23, blood pressure, urinary catecholamines and urinary aldosterone as a surrogate for activation of the renin–angiotensin–aldosterone system (RAAS) [38]. Vice versa, angiotensin II administration to *Hyp* mice led to an increase in FGF23 levels, blood pressure and left ventricular hypertrophy [43]. These findings suggest a vicious circle of phosphate substitution in XLH, i.e. phosphate supplementation further stimulates FGF23 secretion from bone, which in turn activates the RAAS and further increases blood pressure in patients on treatment with phosphate supplements and active vitamin D. The lack of left ventricular hypertrophy in most previous studies in XLH patients on treatment with phosphate supplements and active vitamin D, despite documented elevated blood pressure, may at least partly be related to the cardioprotective properties of concomitant treatment with active vitamin D, as vitamin D is a negative endocrine regulator of the RAAS [44].

The present study has some limitations. Firstly, this was not a randomized-controlled study allowing the best possible investigation of blood pressure values in XLH patients on treatment with phosphate supplements and active vitamin D versus burosumab. In our real-world study, the phenotype in children remaining on treatment with phosphate supplements and active vitamin D was somehow milder compared to those primarily started on or switched to burosumab. However, the milder phenotype and the relatively low phosphate doses probably led to an underestimation of the blood pressure differences in the two groups as treatment with phosphate supplements and active vitamin D was shown to increase FGF23 levels. Secondly, we cannot rule out that some of the elevated blood pressure values in the present study are due to the white coat phenomenon. This would be supported by the fact that isolated systolic hypertension often occurs due to the white coat effect [45]. However, diastolic blood pressure values were also elevated in our cohort and we tried to counteract this problem by using the median of 1–6 blood pressure measurements per patient for our analyses.

In conclusion, systolic and diastolic blood pressure values as well as BMI were found to be significantly elevated in children with XLH compared to healthy children, and the prevalence of arterial hypertension and obesity were significantly increased in XLH patients compared to the general pediatric population. Moreover, blood pressure values were significantly associated with the degree of obesity in our patient cohort. Therefore, pediatric patients with XLH should be monitored regularly for cardiovascular risks.

## Supplementary Information

Below is the link to the electronic supplementary material.

Supplementary file1 (DOCX 18 KB)

## Data Availability

Some or all datasets generated and/or analyzed during the current study are not publicly available but are available from the corresponding author on reasonable request.
