# Supplementary material for: Office Blood Pressure and Obesity in Children with X-Linked Hypophosphatemia
Source: Calcif Tissue Int. 2025 Mar 28;116(1):56. doi: 10.1007/s00223-025-01363-z (PMC11953092; doi:10.1007/s00223-025-01363-z)
Supplement: Supplementary file 1 — Supplementary file1 (DOCX 18 KB) [file 223_2025_1363_MOESM1_ESM.docx]

| **Characteristics** | **All patients** | **Patients with hypertension** | **Patients without hypertension** | ***p*** |
| --- | --- | --- | --- | --- |
| n | 128 (100) | 29 (22.7) | 99 (77.3) | n.a. |
| Female, n | 72 (56) | 17 (58.6) | 55 (55.5) | 0.770 |
| Age at diagnosis, years | 1.1 (0.2 - 2.5) | 1.0 (0.0 - 3.1) | 1.1 (0.2 - 2.5) | 0.724 |
| Age at observation, years | 10.3 (5.4 -14.1) | 9.4 (5.3 - 13.8) | 10.4 (5.3 - 14.1) | 0.918 |
| Height, cm | 129.6 (105.1 – 149.0) | 124.8 (103.5 - 149.3) | 130.2 (105.1 - 149.4) | 0.923 |
| Height, z-score | -1.92 (-2.65 to -1.22) ^a^ | -2.13 (-2.93 to -1.22) ^a^ | -1.91 (-2.55 to -1.22) ^a^ | 0.769 |
| Body weight, kg | 30.1 (18.5; 51) | 27.3 (18.1 - 56.7) | 30.1 (18.6 - 50.0) | 0.903 |
| Body weight, z-score | -0.70 (-1.29 - 0.12) ^a^ | -0.69 (-1.66 - 0.31) ^a^ | -0.69 (-1.26 - 0.09) ^a^ | 0.916 |
| P+vit.D treatment, years | 7.95 (2.44 - 11.03) | 3.9 (0.8 - 9.2) | 9.2 (3.0 - 11.5) | 0.218 |
| Burosumab, years | 2.28 (1.14 - 3.20) | 2.1 (1.4 - 3.5) | 2.3 (1.1 - 3.2) | 0.613 |
| Prior P+vit.D treatment, years | 3.10 (0.66 - 8.52) | 2.4 (0.3 - 8.4) | 4.0 (0.8 - 8.5) | 0.567 |
| Phosphorus, mg/kg/day | 21.0 (10.8 - 29.1) | 21.6 (14.6 - 36.5) | 20.9 (9.5 - 27.8) | 0.495 |
| Calcitriol, ng/kg/day | 11.7 (4.0 - 28.0) | 14.0 (3.3 - 41.0) | 11.7 (4.0 - 28.0) | 0.698 |
| Burosumab, mg/kg/14 days | 0.6 (0.4 - 1.1) | 0.59 (0.29 - 1.04) | 0.67 (0.39 - 1.14) | 0.162 |
| Serum phosphate, mmol/l | 1.00 (0.87 -1.13) | 1.00 (0.75 - 1.07) | 1.00 (0.88 - 1.16) | 0.178 |
| Serum phosphate, z-score | -2.78 (-3.57 to -1.99) ^a^ | -3.01 (-3.59 to -2.25) ^a^ | -2.73 (-3.58 to -1.91) ^a^ | 0.369 |
| Serum ALP, U/l | 336 (252 - 432) | 313 (228 - 459) | 346 (252 - 426) | 0.697 |
| Serum ALP, z-score | 1.73 (0.77 - 2.78) ^a^ | 1.74 (0.71 - 3.01) ^a^ | 1.73 (0.86 - 2.68) ^a^ | 0.959 |
| Serum PTH, ng/l | 46.4 (34.6 - 69.8) | 42.3 (30.6 - 64.4) | 49.2 (35.8 - 71.7) | 0.174 |
| Serum PTH, z-score | 1.24 (0.44 - 2.35) ^a^ | 0.98 (0.09 - 2.13) ^a^ | 1.40 (0.53 - 2.43) ^a^ | 0.174 |
| 25OHD, ng/ml | 25.3 (20.0 - 32.8) | 21.4 (17.0 - 32.4) | 23.9 (20.3 - 29.3) | 0.423 |
| 25OHD <30 ng/ml, n | 81 (63.3) | 13 (44.8) | 51 (51.5) | 0.422 |
| 25OHD <20 ng/ml, n | 29 (22.7) | 7 (24.1) | 13 (13.1) | 0.113 |
| eGFR, ml/min/1.73m^2^ | 127 (106 - 144) | 124 (102 - 132) | 128 (106 - 145) | 0.535 |

**Supplementary table 1.** Comparison of clinical characteristics of 128 pediatric XLH patients with and without hypertension

Data is presented as median (interquartile range) or n (%), *p* values were calculated using unpaired Mann-Whitney test or Chi-squared-Test, respectively. ^a^ p < 0.001 versus healthy controls; P+vit.D, phosphate supplements and active vitamin D; eGFR, estimated glomerular filtration rate; ALP, alkaline phosphatase; PTH, parathyroid hormone.
